# Commonly Rare and Rarely Common: Comparing Population Abundance of Invasive and Native Aquatic Species

**DOI:** 10.1371/journal.pone.0077415

**Published:** 2013-10-23

**Authors:** Gretchen J. A. Hansen, M. Jake Vander Zanden, Michael J. Blum, Murray K. Clayton, Ernie F. Hain, Jennifer Hauxwell, Marit Izzo, Matthew S. Kornis, Peter B. McIntyre, Alison Mikulyuk, Erika Nilsson, Julian D. Olden, Monica Papeş, Sapna Sharma

**Affiliations:** 1 Center for Limnology, University of Wisconsin-Madison, Madison, Wisconsin, United States of America; 2 Department of Ecology & Evolutionary Biology, Tulane University, New Orleans, Louisiana, United States of America; 3 Departments of Plant Pathology and Statistics, University of Wisconsin-Madison, Madison, Wisconsin, United States of America; 4 Department of Biology, North Carolina State University, Raleigh, North Carolina, United States of America; 5 Science Services, Wisconsin Department of Natural Resources, Madison, Wisconsin, United States of America; 6 School of Aquatic and Fishery Sciences, University of Washington, Seattle, Washington, United States of America; University of Pretoria, South Africa

## Abstract

Invasive species are leading drivers of environmental change. Their impacts are often linked to their population size, but surprisingly little is known about how frequently they achieve high abundances. A nearly universal pattern in ecology is that species are rare in most locations and abundant in a few, generating right-skewed abundance distributions. Here, we use abundance data from over 24,000 populations of 17 invasive and 104 native aquatic species to test whether invasive species differ from native counterparts in statistical patterns of abundance across multiple sites. Invasive species on average reached significantly higher densities than native species and exhibited significantly higher variance. However, invasive and native species did not differ in terms of coefficient of variation, skewness, or kurtosis. Abundance distributions of all species were highly right skewed (skewness>0), meaning both invasive and native species occurred at low densities in most locations where they were present. The average abundance of invasive and native species was 6% and 2%, respectively, of the maximum abundance observed within a taxonomic group. The biological significance of the differences between invasive and native species depends on species-specific relationships between abundance and impact. Recognition of cross-site heterogeneity in population densities brings a new dimension to invasive species management, and may help to refine optimal prevention, containment, control, and eradication strategies.

## Introduction

Invasive species are recognized as leading drivers of environmental change, and can produce significant economic and ecological impacts [1]. Recently, Davis et al. [2] argued that conservationists, scientists, and the general public hold a pervasive bias against non-native species, and that species should be judged based on their impact rather than their place of origin. The debate that has followed (e.g., [3],[4]) highlights the importance of understanding variation in the impacts of invasive species for both science and resource management, and serves as a reminder of the need to question and empirically test assumptions about invasive species.

Invasion biology research has aimed to elucidate general patterns, sometimes at the cost of overlooking important sources of heterogeneity [5]. It has long been recognized that only a small fraction of introduced species will establish, spread, and cause impacts [6]. As a result, identifying species likely to cause ecological or economic impacts and predicting locations that are likely to be most vulnerable are important goals of invasive species research and management [7]–[9]. Although in principle it is recognized that abundance (and therefore impact) of invasive species varies among sites, few attempts to quantify relative impacts of invasive species incorporate spatial variability in abundance [8]. Instead, species invasions are largely seen as binary phenomena, where an introduced species is either invasive or not (e.g., [7]); and a location is either invaded (or invasible) or not (e.g., [9]; but see [10]). Consequently, most invasive species monitoring and databases emphasize occurrence rather than abundance (Table S1 in File S1). Admittedly, documenting occurrence is logistically more feasible than documenting abundance, particularly across large spatial scales. However, we may miss important insights by ignoring variability among sites in invasive species abundance.

In contrast to the largely dichotomous paradigm of invasive species research noted above, ecologists have long documented that the abundance of many species tends to be low at most locations and high only in a few; that is, frequency distributions of abundance are right skewed [11], [12]. This empirical pattern of right-skewed abundance distributions is observed across a wide range of taxonomic groups and spatial scales, and is considered central to ecological theory [13], [14]. Despite the importance of invasive species abundance in determining impacts [15], [16], virtually no studies have examined whether invasive species follow this same pattern of right-skewed frequency distribution of abundance (but see [17]).

Understanding invasive species abundance distributions and how they compare to those of native species is important for analysis and management of species invasions. Competing predictions are possible based on different lines of reasoning. On one hand, invasive species are often considered inherently different from native species [18], in terms of both biological traits (e.g., high reproductive potential [7]), and community interactions (e.g., lack of natural enemies [19]), leading some to define invasive species as non-indigenous species that become abundant or ‘dominant’ [7], [20]. This view implies that invasive species commonly (or always) reach high densities where they establish populations, which would lead to higher mean abundances with less right-skewed distributions. On the other hand, proposed ecological mechanisms behind right-skewed abundance distributions such as differences in niche suitability among sites [11], [21] likely apply equally to species in both their native and introduced ranges. Recent attempts to quantify differences between invasive and native species abundance have found subtle or no differences between the groups (e.g.,[22], [23]), indicating that perhaps invasive species play by the same ecological rules, following right-skewed abundance frequency distributions similar to those of native counterparts.

In this analysis we compare patterns of population abundance for native and invasive aquatic species, collected simultaneously using the same methods. For each species included in our study, we obtained abundance estimates across many sites. We characterized the abundance distribution of each species in terms of its statistical moments (i.e., mean, variance, skewness, and kurtosis), with the objective of determining whether abundance distributions of invasive and native species differed in terms of these statistical moments. We conclude by discussing the implications of invasive species abundance distributions for ecological theory and the management of invasive species.

## Methods

### Ethics statement

All data existed prior to the initiation of this study and include our own data collected for other purposes, public data collected by management agencies, and published literature sources. Data sources and sampling methods are described in detail in Supplementary Methods S1 in File S1. For data collected by the authors, all animals were captured following protocols approved by the relevant Institutional Animal Care and Use Committee (IACUC), and permits for sampling were obtained from the appropriate authorities (noted where appropriate in Supplementary Methods S1 in File S1).

### Data collation and analysis

**Figure 1:**
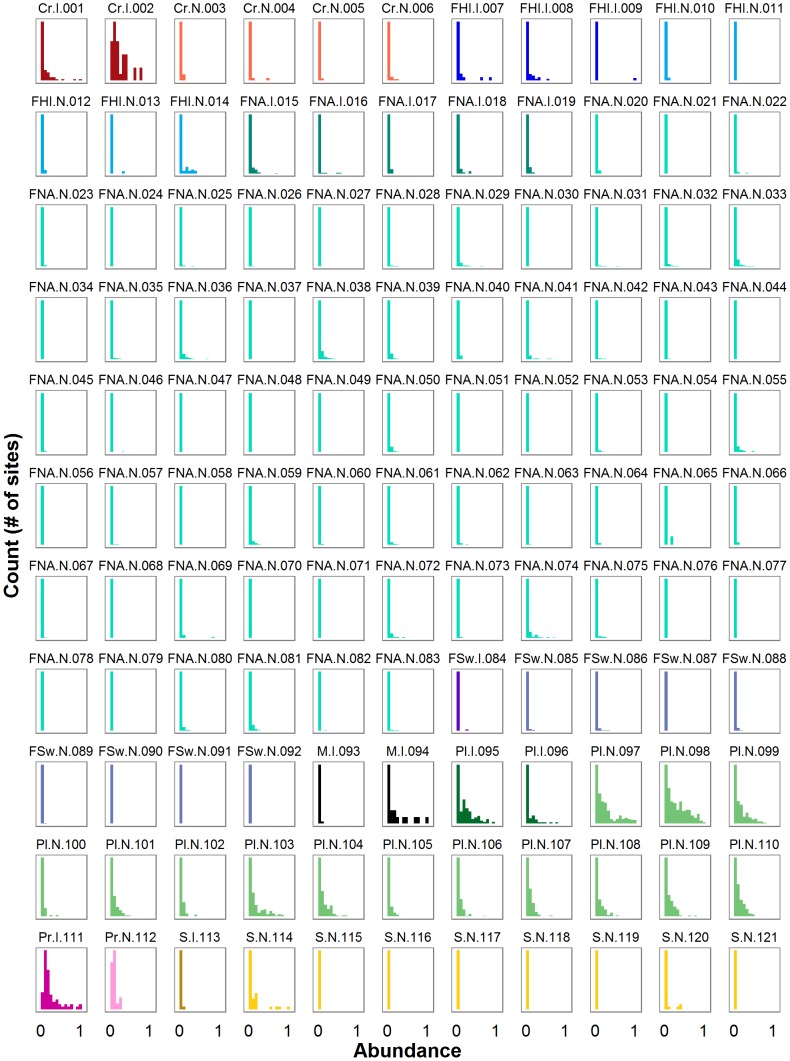
Abundance distributions for each species used in this analysis. Labels are coded as follows: taxonomic group abbreviation. Origin. Species ID, where taxonomic group codes are Cr = Crayfish, FHI = Hawaiian fishes, FNA = North American fishes, FSw = Swedish fishes, M = Mussel, Pl = Plant, Pr = Prawn, and S = Snail; origin codes are I = Invasive and N = Native; see Table S3 in File S1 for species identities. Colors correspond to taxonomic groups and in every group the darker shade corresponds to invasive species in that group. The x-axis scale shows standardized abundance (proportion of taxonomic group-level maximum abundance) and ranges from 0 to 1; the y-axis scale shows the number of sites falling into each abundance class and varies by species to accommodate different numbers of observations (sites). Note that all abundance values are greater than zero.

To examine variability in abundance across sites, we required abundance records for native and invasive species in multiple locations. We compared cross-site abundance distributions of 17 invasive and 104 native species of aquatic plants, invertebrates (crayfish, mussels, prawns, snails), and fishes from three distinct geographic regions (Hawai‘i, North America, and Europe; Figure 1). We grouped species into eight taxonomic/geographical categories (hereafter taxonomic groups) that were sampled using comparable methods: Crayfish, Hawaiian fish, North American fish, Swedish fish, Mussels, Plants, Prawns, and Snails. The invasive species included in this analysis are defined as “species that are non-native[…]to the ecosystem under consideration and whose introduction causes or is likely to cause economic or environmental harm or harm to human health” [24] and are classified as “invasive” or nuisance species by authorities in each study region (Table S2 in File S1). All invasive species included here have been present for at least 19 years in the invaded study region (Table S2 in File S1) suggesting that their populations should no longer reflect any transient expansion dynamics that could prevail during the early stages of invasion. Our analysis included 24,033 non-zero density records, with 20 to 1,252 site-level abundance records per species (Table S3 in File S1). To facilitate comparisons among species abundance reported in different units, abundances were standardized to range from zero to one by dividing each raw abundance value by the maximum observed value within each taxonomic group.

To characterize abundance distributions, we calculated the first four statistical moments (i.e., mean, variance, skewness, and kurtosis) of the abundance distribution of each species. Preliminary analysis showed that mean and variance were highly correlated; we also calculated coefficient of variation (CV) to compare variation independent of this correlation. Moments were natural log transformed and differences in moments of invasive and native species abundances were estimated using a multi-level modeling approach [25] with origin (invasive or native) as a fixed effect, and taxonomic group as a random effect. Effects are reported as restricted maximum likelihood (REML) estimates of fixed effects from the lme4 package [26] in R v2.15.1 [27]. Confidence intervals were calculated as Bayesian highest posterior density (HPD) intervals of parameter estimates generated from 10,000 Markov Chain Monte Carlo (MCMC) simulations using the languageR package, which incorporates variation from random effects [28]. Differences in moments based on species origin (i.e., differences between invasive and native species) were considered statistically significant if the 95% HPD intervals of estimated difference between native and invasive species did not include zero [25], [29]. Adjusted R^2^ values for mixed effects models were calculated as the likelihood ratio test R^2^ [30], [31].

We generated empirical cumulative distributions (ECD's) for invasive and native species abundance overall and within each taxonomic group. For these distributions we disregarded species identity and constructed ECD's using abundances of all invasive and all native species in each taxonomic group. We then identified the median abundance (ECD = 0.5) of invasive species within each group, and compared the ECD value of native species for the same abundance value. This allowed us to compare the proportion of sites containing native species at abundances equal to or above the median invasive species abundance.

## Results

**Figure 2:**
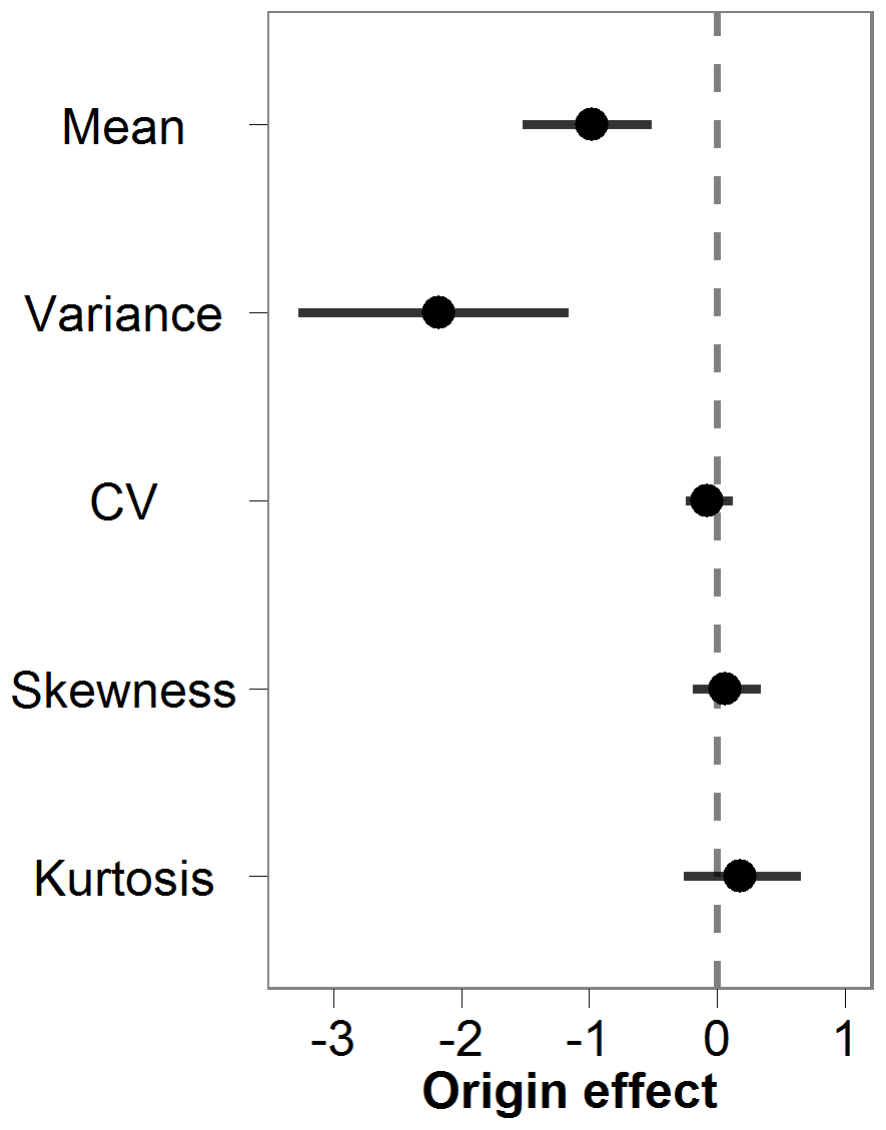
Effect size of origin (invasive vs. native status) on distributional parameters. Effects are presented as the restricted maximum likelihood (REML) estimate of the difference on the natural log scale between invasive and native species (or the natural log of the ratio of invasive:native species values). Bars are 95% highest probability density interval from Markov-chain Monte Carlo (MCMC) resampling; bars that do not overlap zero (dashed line) represent significant differences between invasive and native species.

Abundance distributions of all species were highly right-skewed (skewness > 0; Table S3 in File S1), regardless of invasive status; all species occurred at low densities in the vast majority of sites where they were documented (Figure 1). Invasive species on average reached significantly higher densities than native species and exhibited significantly higher variance (Table 1; Figure 2). However, invasive and native species did not differ in terms of CV, skewness, or kurtosis (Table 1, Figure 2). The absolute difference in mean abundance of invasive and native species was small (Table 1). Mean standardized abundance of invasive species was 0.06 and that of native species was 0.02 on the standardized abundance scale. In other words, the average abundance of invasive and native species was 6% and 2%, respectively, of the maximum abundance observed within a taxonomic group.

**Table 1:** Model results for hierarchical models of statistical moments.

Moment	R^2^	Fixed effect	Estimate	Upper HPD	Lower HPD	Random effect	Variance
Mean	0.47	Intercept	−2.81	−2.15	−3.46	Taxa	1.05
		Origin	−0.98	−0.51	−1.52	Residual	0.76
Variance	0.33	Intercept	−4.74	−3.52	−6.09	Taxa	2.22
		Origin	−2.18	−1.16	−3.28	Residual	3.65
CV	0.16	Intercept	0.45	0.68	0.24	Taxa	0.08
		Origin	−0.08	0.12	−0.24	Residual	0.10
Skewness	0.26	Intercept	0.94	1.27	0.61	Taxa	0.20
		Origin	0.06	0.34	−0.19	Residual	0.22
Kurtosis	0.26	Intercept	2.33	2.91	1.79	Taxa	0.56
		Origin	0.18	0.66	−0.26	Residual	0.66

The intercept represents the restricted maximum likelihood (REML) estimate of the value for invasive species, and the origin effect is the difference between invasive and native species values on the natural log scale. Upper and lower highest probability density (HPD) intervals are the 95% confidence intervals of the fixed effects estimates generated from Markov-chain Monte Carlo resampling. Random effects and their explained variance are also presented for each model, where the taxa effect is the variance attributable to differences among taxonomic groups in statistical moments.

We were able to compare abundance distributions of three species in their native and invaded ranges: brook trout (*Salvelinus fontinalis*), brown trout (*Salmo trutta*), and signal crayfish (*Pacifastacus leniusculus*). Although small sample size precludes formal statistical inference, no consistent patterns in statistical moments of species in their invaded compared to native ranges were evident (Table S3 in File S1). Two of three species (brown trout and signal crayfish) exhibited patterns similar to those observed in the full dataset: higher mean abundance and variance in their invaded range. However, two of three species (brook trout and brown trout) exhibited patterns distinct from those observed overall; these species were more right-skewed and exhibited higher kurtosis in their invaded range.

**Figure 3:**
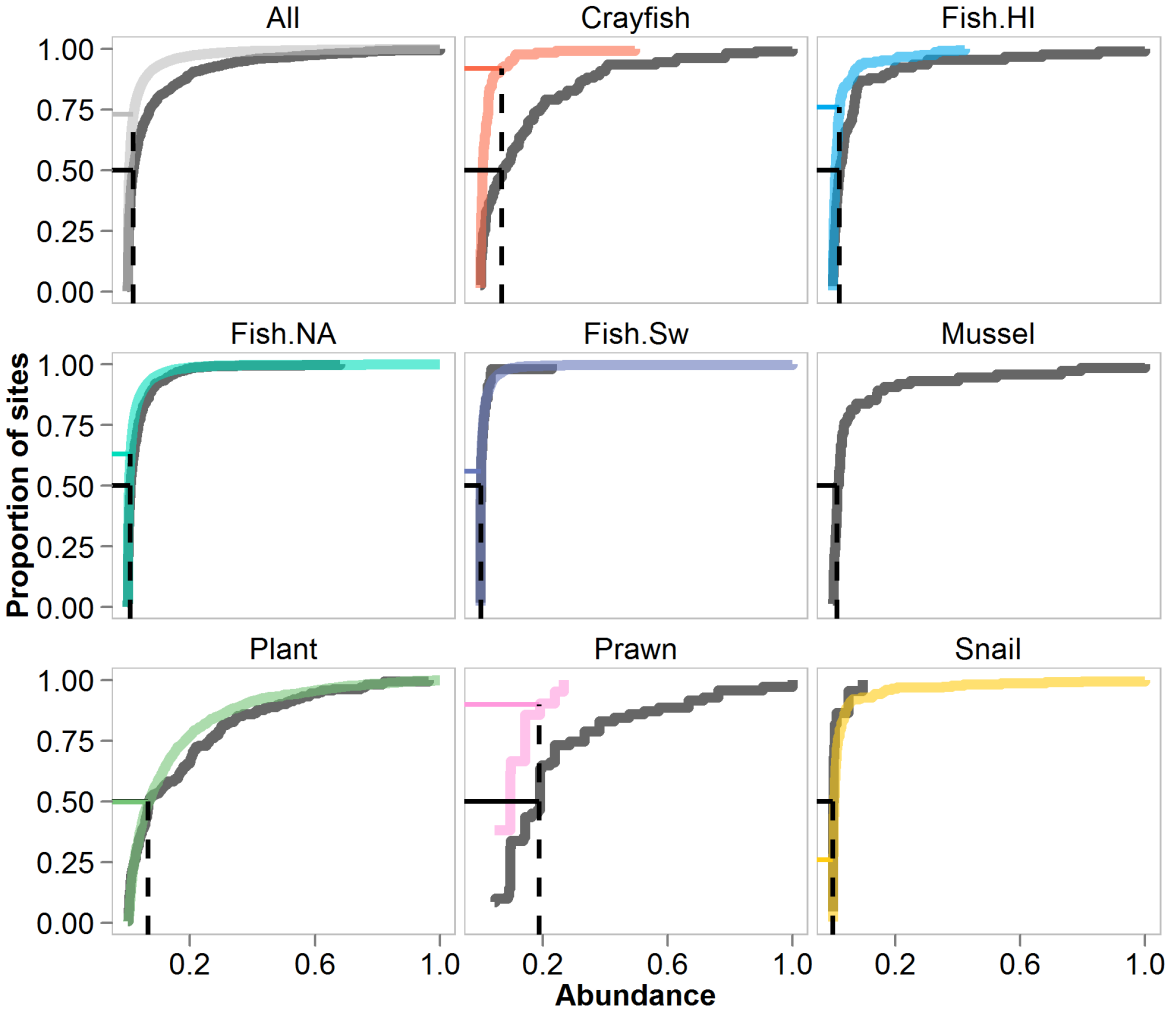
Empirical cumulative distributions (ECD) of invasive and native species. ECDs for invasive (dark grey) and native (colors) species abundance for each taxonomic group. The x-axis is standardized abundance, calculated as the proportion of the maximum abundance observed within a taxonomic group. The probability of finding a species from a given taxonomic group at or below the corresponding x-axis value is plotted on the y-axis. Median abundance for invasive species of each taxonomic group (ECD = 0.5) occurs where the cumulative distribution crosses the black horizontal line, and this median abundance value for invasive species is shown by the vertical black dashed line. The ECD value for native species corresponding to median invader abundance is indicated with a colored horizontal line, and represents the probability of finding native species of each taxonomic group at or below the median invasive value.

Empirical cumulative frequency distributions of native and invasive species abundance for each taxonomic group were similar, but not identical (Figure 3). The maximum observed abundance value within a taxonomic group was that of a native species in four taxonomic groups - North American fish, Swedish fish, aquatic plants, and snails - but this pattern did not lead to higher mean abundance of native species (Figure 2). In six taxonomic groups, native species were present at densities greater than the median density of invasive species at fewer than 50% of sites (Figure 3). This trend was most prominent for crayfish and prawns; native species were present at or above invasive species median abundances at only 8% and 10% of sites, respectively. Snails showed the opposite trend, with native species present at or above invasive species median abundance at 74% of sites.

## Discussion

**Figure 4:**
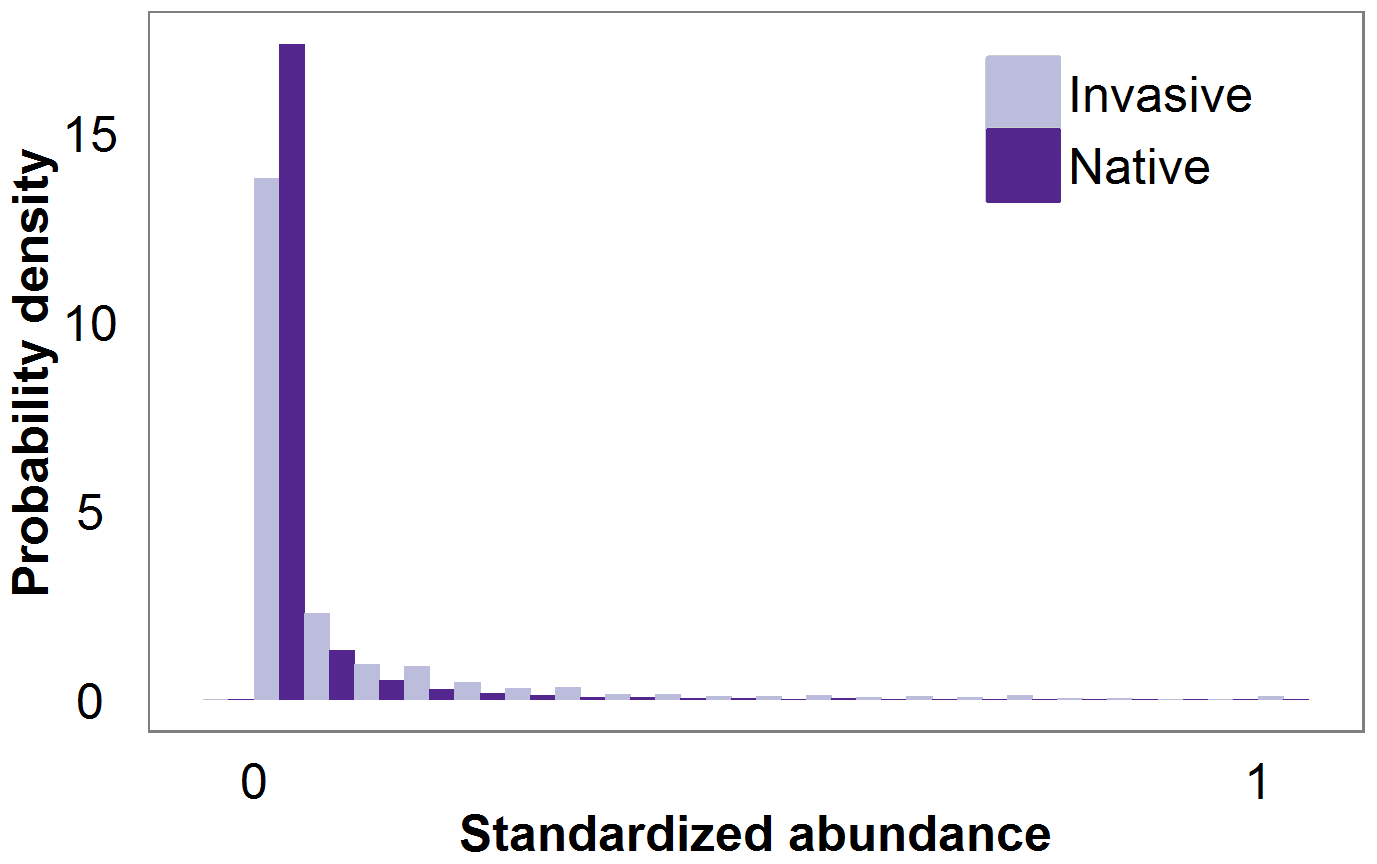
Abundance distributions of all invasive and all native species combined. Probability density of standardized abundance (proportion maximum abundance observed within a taxonomic group) for invasive (light purple) and native (dark purple) species, with all species combined. Abundance values are grouped into 0.05 bins.

We identified subtle differences in the abundance distributions of invasive and native aquatic species from a wide range of locations and taxonomic groups. On average, invasive species reached higher abundances than native counterparts in the same region; mean abundance of invasive species was three times greater than the mean abundance of native species. The higher observed abundance of invasive species in this study may in part be driven by biased data collection. Invasive species that typically establish small populations and have low ecological or environmental impact are not well represented in existing data sets and/or the literature. These species are generally considered lower priority for study by management and regulatory authorities. Despite this potential bias, absolute differences between mean abundance of invasive and native species were small (a difference of 0.04 on the standardized abundance scale). Indeed, variability in distributional parameters was high, and for most taxonomic groups (4 of 7) a native species was responsible for the maximum abundance observed within the group (Figure 3). The subtle difference between invasive and native species abundance distributions can be visualized by plotting standardized abundance distributions for all native and all invasive species combined (Figure 4). Although native species are more likely than invasive species to occur in the lowest abundance class (0–0.05 on standardized scale), the vast majority of all species abundances fall within this range regardless of invasive status.

The biological significance of differences between invasive and native species abundance distributions depend on species-specific relationships between density and ecological impact. Unfortunately, knowledge of such relationships is lacking for even the most notorious invaders [32]. Although some species may exert ecological effects disproportional to their abundance (so-called “keystone species”; [33]), the impact of an invasive species is generally positively correlated with its abundance [15], [16], [34]. Our results therefore suggest that the impact of most invasive species will be high in a small number of locations, and relatively low in the majority of invaded sites. However, in the absence of data relating density to impacts, it is impossible to know how impacts will scale with relative abundance– that is, whether a site in which a species reaches 2% of the maximum observed density within a taxonomic group will experience 2% of the maximum observed impact. Similarly, the differences between invasive and native species are likely more important for certain taxonomic groups than others, particularly when invader density is related to impact via a non-linear threshold response [35]. For example, negative effects of one invasive crayfish species occur at densities ≥9•trap^−1^ [36], or 0.28 on the standardized abundance scale. Our analysis shows that invasive crayfish exceed this threshold in ∼17% of sampled locations, while native crayfish do so in less than 1% of locations (Figure 3). Thus, the differences in the abundance distributions of invasive and native crayfish could translate into very real differences in ecological impacts on the landscape scale if the per capita effects of crayfish species are similar. In contrast, the impacts of one invasive snail species are highest at densities above 2 m^−2^ [37], or 0.01 on the standardized abundance scale. Native snails exceed this threshold in approximately 35% of sites, while invasive snails do so in only ∼15% of sites, suggesting that the ecological effects of invasive snails may be less than those of natives if per capita impacts are similar. Quantifying per capita effects of invasive species (e.g., [38]), as well as the relationship between invasive species abundance and impact (e.g., [10],[35]), is a requisite to interpreting the biological significance of the statistical patterns identified here. Unfortunately, such thresholds have not been identified in any systematic way for most invasive species, and it remains unclear whether generalized thresholds for impact could be identified across diverse groups.

The observed differences in mean abundance of invasive and native species do not necessarily reflect fundamental ecological differences between them. Widespread species tend to be on average more abundant than species restricted to small ranges, driven by higher maximum abundances [39]. Invasive species by definition have spread outside their native range and most are increasing their range size; higher abundances are therefore expected as their range size increases. Indeed, the relationship between range size and abundance does not differ for invasive and native British bird species, but similar to our findings, invasive species reach higher maximum densities than native counterparts [17]. Other studies have demonstrated that invasive plants rarely reach high densities [40] and the majority of invasive species do not reach higher abundances in invaded compared to native ranges [22], [23], supporting the idea that invasive and native species do not follow fundamentally different distributional patterns. However, the results of our study and that of Labra and colleagues [17] suggest invasive species tend to fall toward the high end of the observed range of abundance-distribution relationships. Identifying the mechanisms explaining such patterns is a fruitful area of current research (e.g., [41]), and the existence of similarities as well as differences in invasive and native species could provide insight into the forces behind these widespread ecological patterns [42].

Every invasive species in this analysis occurred at low population densities in the majority of invaded sites (Figure 1), a finding that is notable given the perceived pervasiveness of these species. In fact, invasive and native species abundance distributions were similarly right-skewed. Low abundances are expected at the edges of species ranges, in areas where a species' niche requirements are not fully met or where it has not yet dispersed [12], [43]. Although the mechanisms driving right-skewed species abundance distributions are the subject of debate [13], [42] and outside the scope of this analysis, the similarly right-skewed distributions of invasive and native species abundance observed here support the idea that invasive and native species are governed by similar ecological processes. The highly right-skewed abundance distributions observed for invasive species could reflect management actions rather than natural patterns in cases where control measures effectively reduce invasive species abundances at most sites. However, only two invasive species in our dataset have been subject to widespread management (sea lamprey, *Petromyzon marinus*; and Eurasian water milfoil; *Myriophyllum spicatum*), and abundance distributions of both managed species are less right-skewed than the majority of invasive species (*skewness_sea lamprey_* = 2.01; *skewness_milfoil_* = 1.23; median skewness of all invasive species = 2.73). Thus, there is no evidence from our analysis that the propensity of invasive species to be present at low abundance arises from control measures.

Another potential explanation for the right-skewed abundance patterns of invasive species is differences among invaded sites in time since species introduction. Temporal variation in species abundance has been shown to generate right-skewed abundance patterns similar to those observed across large spatial scales [44], and many invasive populations exhibit some sort of time lag, or extended period of low abundance [45]. It is impossible to ascertain the time since invasion for each of the over 24,000 individual sites included in this analysis. However, we attempted to decrease the influence of lagged temporal patterns in abundance by restricting our analysis to species that have been present in the study region for at least 19 years (Table S2 in File S1). Several invasive species in this study established nearly a century or more ago, and exhibit similar abundance distributions to those that established more recently, suggesting that right-skewed abundance distributions of invasive species are not an artifact of time lags in population increase. Although this does not rule out the possibility that temporal variability in the abundance of invasive populations is responsible for observed abundance distributions, our results demonstrate that on the temporal scales most relevant to management (years to decades), invasive species exhibit highly right-skewed distributions.

The fact that high density populations of invasive species exist in a small number of “hot spots” has important management implications. Sophisticated modeling techniques are used in risk assessments that aim to identify which species are likely to become invasive (e.g., [46], [47]) and which locations are likely to be invaded (e.g., [48]–[50]), but rarely make predictions about variability in abundance of invasive species. Our results suggest that invasive species control and prevention would benefit from a more nuanced approach that also considers variation in species abundance among invaded sites. For instance, transmissions of infectious diseases follow highly right-skewed distributions similar to species abundances, and the effectiveness of disease control is improved when this heterogeneity is accounted for by predicting the identity of the most potentially infectious individuals and focusing prevention and containment efforts on them prior to a disease outbreak [51]. By analogy, landscape-scale invasive species containment efforts might be enhanced by focusing on the small percentage of “hot spots” containing the majority of individuals of a given invasive species, which would effectively reduce the likelihood of spread to additional sites in the long term. Moreover, because prevention is the most effective tool for combating the negative effects of invasive species [52], the ability to predict which locations are likely to support high densities of invasive species would be useful for targeting prevention so as to minimize impacts. The optimal strategy for invasive species management will be context-dependent, but explicitly considering heterogeneity in invasive species abundance may help guide strategies for invasive species management.

## Conclusion

Heterogeneity in the abundance of invasive species known to cause ecological and economic impacts has received surprisingly little attention. Our finding that aquatic invasive species exist at low densities in most locations where they occur runs counter to the perception of invasive species as those that are abundant or dominant wherever they establish [7], [20]. Both invasive and native species are present in low densities in most locations, supporting the notion that applying general ecological and analytical principles to invasive species will advance understanding more so than treating invasions as idiosyncratic occurrences [53]. At the same time, our finding that invasive species are capable of reaching higher average densities than native species has important implications for invasive species impacts and their management, assuming that ecological impact scales with the population density of invasive species (see also [4]). By recognizing the patchiness of invasive species abundance, our results highlight opportunities to improve prevention, control, and eradication strategies.

## Supporting Information

File S1 Supplementary information, including Supplementary Methods S1 describing in detail the sampling methodology for each group; Table S1 providing examples of invasive species databases that provide presence-absence data only; Table S2 listing the invasive species included in this analysis, their impacts, and year of invasion; and Table S3 listing all species included in this analysis with their associated statistical moments.
(DOCX)
