# Supplementary material for: Commonly Rare and Rarely Common: Comparing Population Abundance of Invasive and Native Aquatic Species
Source: PLoS One. 2013 Oct 23;8(10):e77415. doi: 10.1371/journal.pone.0077415 (PMC3806751; doi:10.1371/journal.pone.0077415)
Supplement: File S1 — Supplementary information, including Supplementary Methods S1 describing in detail the sampling methodology for each group; Table S1 providing examples of invasive species databases that provide presence-absence data only; Table S2 listing the invasive species included in this analysis, their impacts, and year of invasion; and Table S3 listing all species included in this analysis with their associated statistical moments. (DOCX) [file pone.0077415.s001.docx]

**Supplementary Material**

*Supplementary Methods S1: Data collection and collation*

To control for sampling methodology and allow comparisons among native and invasive species, we only included data where both invasive and native species from a taxonomic group were sampled using the same methods across multiple sites. Exceptions were made to include zebra mussel (*Dreissena polymorpha*) data from its invaded range in both Europe and North America. Zebra mussel data were mainly obtained from a meta-analysis [1] which compiled data from 55 European and 13 North American sites from 1959-2004. Additional densities from North America were compiled from multiple sources [2-5]. All zebra mussel records were presented as number per m^2^ and are from their invaded range; we did not include native mussel data. For the purposes of this analysis, North American and European zebra mussel data are treated separately and counted as separate “species” as they represent two distinct samples of abundance distributions.

Crayfish data were obtained from multiple sources. Crayfish were collected in Wisconsin, USA from lakes in the Northern Highlands Lake District during summers of 2002-2010 following the protocol for crayfish collection described at <http://lter.limnology.wisc.edu/>; briefly, ~30 gee-style minnow traps were modified for crayfish sampling, baited with beef liver, and set overnight in 1-3 m water. Crayfish were sampled in Wisconsin streams tributary to Lake Michigan by M. Kornis (unpublished data) from 2007-2010 using 10 gee-style minnow traps per site baited with chicken livers and set overnight. All Wisconsin crayfish were captured following protocols approved by the Wisconsin Department of Natural Resources (WDNR; Scientific Collection Permits SCP-SCR-001-0004, NOR-SCP-292-0410, SCP-NOR-292-2011). Lakes were accessed via public access points. Swedish crayfish were sampled in streams of southern Sweden from 2001-2003 using 30 minnow traps baited with frozen fish as described in [6]. Swedish crayfish were sampled in lakes in August of 2001 using 20 transects containing 5 traps each set perpendicular from shore, baited with frozen fish and set overnight as described in [6] and followed Swedish ethical and sampling guidelines. Permission was obtained from landowners and fishing rights owners prior to sampling. Washington crayfish were captured following protocols approved by the Washington Department of Fish and Wildlife (Scientific Collection Permits 08-344, 09-317, 11-227). Crayfish were collected from 100 lakes in the Puget Sound Lowlands region of Washington State, USA between 2007 and 2009 from mid-June to early October of each year using methods described in [7]. At each lake, the investigators set 20 minnow traps baited with fish-based dog food. Traps were deployed in four clusters of five traps each and recovered the following day. All crayfish densities are presented as number per trap per day.

North American fish data included Wisconsin stream fishes and larval lampreys collected from Great Lakes tributaries in the United States and Canada. The majority of Wisconsin stream fish data were obtained from surveys conducted by employees of the WDNR in the course of their fish management and research activities, and thus were exempt from permit requirements. Fish were collected from streams throughout the state from 2005-2010 using either a backpack or towboat electrofisher with pulsed DC current in wadeable (<1m depth) streams for a minimum of 15 minutes. All fish collected in these samples were released alive. For Wisconsin trout species, locations sampled within 10 years following a stocking event of that species were excluded. Additional fish samples in Wisconsin were collected as described in [8] under Scientific Collectors Permits SCP-NER-167, SE-FH-09-07a, SE-FH-04-08, SE-FH-12-09, SE-FH-04-10, and SE-FH-01-11. The accessed land was mainly public, but private land was occasionally accessed with verbal permission from the land owner. Protected species were not targeted by sampling, and all individuals of non-round goby species were released alive at the site of capture. Round gobies that were retained for further analyses were retained with permission from the Wisconsin DNR, and were euthanized humanely in accordance with animal care protocols. Lamprey data were collected from 2008-2010 from Great Lakes tributaries using ABP-2 backpack electroshockers (University of Wisconsin, Engineering Technical Services, Madison, Wisconsin) following standardized methods as a part of the sea lamprey assessment program of the United States Fish and Wildlife Service and Department of Fisheries and Oceans, Canada [9,10]. Native lampreys were released and sea lampreys were euthanized according to the protocol for each agency. North American fish densities are presented as number per minute of sampling.

Swedish fish data were collected using backpack electrofishing from 1980 to 2010 from streams in Vasterbotten county, northern Sweden, and were obtained from the Swedish Electrofishing REgister (SERS), <http://www.havochvatten.se>, and are reported as number per 100 m^2^ of stream. Hawaiian fish and prawn data were collected by M. Blum, E. Hain, and P. McIntyre (unpublished data) from June 2009 to August 2011, and authorized by the Hawaii Division of Aquatic Resources (Scientific Collectors Permits SAP 2009-52, SAP 2009-60, SAP 2012-14, SAP 2012-15). Between one and six sites were surveyed within each of 40 watersheds located across the five Hawaiian Islands with perennial streams: Hawai’i, Kaua’i, Maui, O’ahu and Moloka’i. At each site, snorkelers surveyed the densities of native and non-native fishes and invertebrates within thirty 1 m^2^ quadrats distributed randomly within a 140 m long reach [11], and are reported as number per m^2^.

Snail data were collected in 2006 from lakes in the Northern Highlands Lake District in Wisconsin as described by [12], and sampling was approved by the WDNR (Scientific Collectors Permit NOR-SCP-292-0406). Snail densities are presented as number per two m^2^. All aquatic plant surveys were conducted by employees of the WDNR in the course of their regular aquatic plant management and research activities and thus exempt from permitting requirements. Aquatic plant data were collected using a systematic grid-based point-intercept sampling methodology to record macrophyte frequency of occurrence in 242 Wisconsin lakes from 2005-2008. Lakes were accessed using public access points and aquatic plant presence/absence was recorded from a boat using a double-sided rake sampler at each point on a sampling grid as described in [13]. Density data are presented as frequency of occurrence, specifically the proportion of sites within lake littoral zone where a species was present.

For all species from all taxonomic groups, if multiple records existed from the same location, we used the most recent record. If replicate samples existed within the same site on the same sampling date, the mean value was used. Data for each species are summarized in Table S3, and raw abundance data for each species are available at <http://lter.limnology.wisc.edu/>.

**Supplementary Tables**

Table S1. Examples of invasive species databases of invasive species impacts and distribution, none of which report invasive species densities or abundances at multiple sites.

| Name | Geographic coverage | Distributional information | URL |
| --- | --- | --- | --- |
| Global invasive species database | Global | Presence and status by country/state | <http://www.issg.org/database/welcome/> |
| National Biodiversity Network (NBN) | United Kingdom | Presence by 100m^2^ grid | http://data.nbn.org.uk/ |
| Non-indigineous species (NIS) base | Global | Varies (no density information) | <http://www.nisbase.org/nisbase/index.jsp> |
| The European network on invasive alien species | Europe | Status by country | <http://www.nobanis.org/> |
| United States Geological Survey (USGS) Aquatic gap analysis program | Parts of United States | Presence by watershed | [http://www.gapanalysis.usgs.gov/gap-analysis/aquatic-gap](http://www.gap.uidaho.edu/species_data.html) |
| USGS Non-indigenous aquatic species (NAS) | United States | Presence by watershed | <http://nas.er.usgs.gov/default.aspx> |

Table S2. Invasive species included in this analysis and their native range, invaded region (used in this study), estimated year of establishment in invaded region, and their ecological impacts. Invasive species are defined as non-native species that are spreading and have demonstrated or perceived ecological impacts. All invasive species included in this analysis have been classified as “invasive” and are restricted by the regional fisheries management agency in their invaded range (Hawai’i for Liberty/Mexican molly, guppy, and Tahitian prawn, [http://www.Hawai’iwatershedatlas.com/index.html](http://www.hawaiiwatershedatlas.com/index.html); Sweden for signal crayfish and brook trout, [www.nobanis.org](http://www.nobanis.org); Wisconsin for all others, <http://dnr.wi.gov/invasives/classification/>).

| **Common name** | **Latin name** | **Native range (Invaded range)** | **Estimated year of invasion** | **Ecological impacts** | **Key references** |
| --- | --- | --- | --- | --- | --- |
| Rusty crayfish | *Orconectes rusticus* | Ohio River valley (Wisconsin lakes and streams) | 1970's [14-16] | Native crayfish, macrophytes, benthic macroinvertebrates, native fishes | [16-18] |
| Signal crayfish | *Pacifastacus leniusculus* | Western United States (Swedish lakes and streams) | 1969 [19] | Native crayfish, macrophytes, benthic macroinvertebrates, amphibians | [20-22] |
| Liberty/ Mexican molly | *Poecilia sphenops* | Central America (Hawaiian streams) | 1922 [23] | Native fishes | [24,25] |
| Guppy | *Poecilia reticulata* | South America (Hawaiian streams) | 1922 [23] | Native fishes | [24-27] |
| Green Swordtail | *Xiphophorus hellerii* | Central America (Hawaiian streams) | 1922 [23] | Native fishes | [24-27] |
| Brown trout | *Salmo trutta* | Europe (Wisconsin streams) | 1887 [28] | Native fishes, invertebrates | [29-31] |
| Common carp | *Cyprinus carpio* | Asia (Wisconsin streams) | 1880's-1890's [32] | Water quality, macrophytes, macroinvertebrates | [33-35] |
| Rainbow trout | *Oncorhynchus mykiss* | Western United States (Wisconsin streams) | 1880 [28,36] | Native fishes, amphibians | [29,37,38] |
| Round goby | *Neogobius melanostomus* | Ponto-Caspian region (Wisconsin tributaries of Lake Michigan) | 1993 [39] | Native fishes, benthic macroinvertebrates | [40-42] |
| Sea lamprey | *Petromyzon marinus* | Atlantic Ocean (Great Lakes tributaries) | 1920's-1940's [43] | Native fishes | [44-46] |
| Brook trout | *Salvelinus fontinalis* | Eastern North America (Swedish streams) | 1891 [47] | Native fishes | [48,49] |
| Zebra mussel | *Dresseina polymorpha* | Ponto-Caspian region (European lakes; North American Lakes) | 1800's (Europe) [1,50]; 1986-1991 (North America) [1,51] | Native mussels, zooplankton, benthic macroinvertebrates, native fishes, water birds, biofouling | [52-54] |
| Curly-leaf pondweed | *Potamogeton crispus* | Europe, Africa, Asia, Australia (Wisconsin lakes) | 1906 [55] | Native plants, nutrient cycling | [56,57] |
| Eurasian water milfoil | *Myriophyllum spicatum* | Europe, Asia, Northern Africa (Wisconsin lakes) | 1962 [55] | Native plants, macroinvertebrates | [58-60] |
| Tahitian prawn | *Macrobrachium lar* | Indo-West Pacific (Hawaiian streams) | 1956 [23] | Native fishes, native macroinvertebrates | [27] |
| Chinese mystery snail | *Bellamya chinensis* | Asia (Wisconsin lakes) | 1931 [61] | Native snails, nutrient cycling, periphyton | [12,61] |

Table S3. All species included in analyses, with corresponding species ID (used for identification in Figure 1), taxonomic group, geographic region from which data were collated, number of sites with non-zero observations (N), and calculated statistical moments based on standardized abundance (proportion of maximum abundance observed within a taxonomic group). Data sources described in supplementary text.

| **Species ID** | **Taxonomic group** | **Common Name** | **Species** | **Origin** | **Region** | **N** | **Mean** | **Variance** | **Skewness** | **Kurtosis** | **CV** |
| --- | --- | --- | --- | --- | --- | --- | --- | --- | --- | --- | --- |
| Cr.I.001 | Crayfish | Rusty crayfish | *Orconectes rusticus* | Invasive | Wisconsin Lakes; Wisconsin streams | 76 | 0.103 | 0.032 | 3.19 | 14.58 | 1.73 |
| Cr.I.002 | Crayfish | Signal crayfish | *Pacifastacus leniusculus* | Invasive | Sweden | 34 | 0.245 | 0.043 | 1.30 | 3.90 | 0.84 |
| Cr.N.003 | Crayfish | Native crayfish | *Procambarus spp., Orconectes spp.* | Native | Wisconsin streams | 32 | 0.025 | 0.001 | 1.44 | 4.22 | 1.00 |
| Cr.N.004 | Crayfish | Northern crayfish | *Orconectes propinquus* | Native | Wisconsin Lakes | 26 | 0.034 | 0.009 | 4.39 | 21.37 | 2.89 |
| Cr.N.005 | Crayfish | Virile crayfish | *Orconectes virilis* | Native | Wisconsin Lakes | 31 | 0.011 | 0.000 | 4.09 | 20.55 | 1.80 |
| Cr.N.006 | Crayfish | Signal crayfish | *Pacifastacus leniusculus* | Native | Washington Lakes | 53 | 0.026 | 0.002 | 3.18 | 13.74 | 1.76 |
| FHI.I.007 | Hawaiian fish | Liberty/ Mexican molly | *Poecilia sphenops* | Invasive | Hawaiian Streams | 22 | 0.100 | 0.048 | 2.77 | 9.17 | 2.19 |
| FHI.I.008 | Hawaiian fish | Guppy | *Poecilia reticulata* | Invasive | Hawaiian Streams | 47 | 0.068 | 0.011 | 2.74 | 11.28 | 1.54 |
| FHI.I.009 | Hawaiian fish | Green swordtail | *Xiphophorus hellerii* | Invasive | Hawaiian Streams | 23 | 0.067 | 0.042 | 4.40 | 20.59 | 3.07 |
| FHI.N.010 | Hawaiian fish | ‘O‘opu nākea | *Awaous guamensis* | Native | Hawaiian Streams | 89 | 0.015 | 0.000 | 3.34 | 16.31 | 1.35 |
| FHI.N.011 | Hawaiian fish | ‘O‘opu akupa | *Eleotris sandwicensis* | Native | Hawaiian Streams | 39 | 0.006 | 0.000 | 1.46 | 4.73 | 0.84 |
| FHI.N.012 | Hawaiian fish | ‘Āholehole | *Kuhlia sandvicensis* | Native | Hawaiian Streams | 36 | 0.023 | 0.001 | 1.35 | 3.65 | 1.16 |
| FHI.N.013 | Hawaiian fish | O'opu naniha | *Stenogobius hawaiiensis* | Native | Hawaiian Streams | 28 | 0.021 | 0.004 | 4.88 | 25.23 | 2.86 |
| FHI.N.014 | Hawaiian fish | 'O'opu nopili | *Sicyopterus stimpsoni* | Native | Hawaiian Streams | 59 | 0.076 | 0.013 | 1.73 | 4.84 | 1.48 |
| FNA.I.015 | North American fish | Brown trout | *Salmo trutta* | Invasive | Wisconsin streams | 201 | 0.042 | 0.005 | 4.57 | 36.16 | 1.69 |
| FNA.I.016 | North American fish | Common carp | *Cyprinus carpio* | Invasive | Wisconsin streams | 103 | 0.019 | 0.006 | 6.14 | 41.48 | 3.98 |
| FNA.I.017 | North American fish | Rainbow trout | *Oncorhynchus mykiss* | Invasive | Wisconsin streams | 43 | 0.018 | 0.001 | 2.12 | 7.15 | 1.52 |
| FNA.I.018 | North American fish | Round goby | *Neogobius melanostomus* | Invasive | Wisconsin streams , | 60 | 0.034 | 0.004 | 3.59 | 16.40 | 1.91 |
| FNA.I.019 | North American fish | Sea lamprey ammocoete | *Petromyzon marinus* | Invasive | North American Great Lakes tributaries | 260 | 0.029 | 0.001 | 2.01 | 7.29 | 1.25 |
| FNA.N.020 | North American fish | American brook lamprey ammocoete | *Lampetra appendix* | Native | North American Great Lakes tributaries | 122 | 0.016 | 0.001 | 2.39 | 8.83 | 1.57 |
| FNA.N.021 | North American fish | Banded darter | *Etheostoma zonale* | Native | Wisconsin streams | 97 | 0.006 | 0.000 | 2.84 | 11.25 | 1.59 |
| FNA.N.022 | North American fish | Bigmouth shiner | *Notropis dorsalis* | Native | Wisconsin streams | 94 | 0.014 | 0.002 | 6.56 | 51.10 | 2.91 |
| FNA.N.023 | North American fish | Black bullhead | *Ameiurus melas* | Native | Wisconsin streams | 250 | 0.005 | 0.000 | 6.48 | 48.15 | 3.08 |
| FNA.N.024 | North American fish | Black crappie | *Pomoxis nigromaculatus* | Native | Wisconsin streams | 116 | 0.002 | 0.000 | 3.94 | 21.52 | 1.37 |
| FNA.N.025 | North American fish | Blacknose shiner | *Notropis heterolepis* | Native | Wisconsin streams | 199 | 0.008 | 0.001 | 10.40 | 126.38 | 3.44 |
| FNA.N.026 | North American fish | Blackside darter | *Percina maculata* | Native | Wisconsin streams | 275 | 0.006 | 0.000 | 4.32 | 25.08 | 1.69 |
| FNA.N.027 | North American fish | Blackstripe topminnow | *Fundulus notatus* | Native | Wisconsin streams | 28 | 0.007 | 0.000 | 4.08 | 19.58 | 2.05 |
| FNA.N.028 | North American fish | Bluegill | *Lepomis macrochirus* | Native | Wisconsin streams | 362 | 0.008 | 0.001 | 7.64 | 79.11 | 2.80 |
| FNA.N.029 | North American fish | Bluntnose minnow | *Pimephales notatus* | Native | Wisconsin streams | 447 | 0.025 | 0.003 | 5.07 | 38.06 | 2.25 |
| FNA.N.030 | North American fish | Bowfin | *Amia calva* | Native | Wisconsin streams | 21 | 0.003 | 0.000 | 1.73 | 5.18 | 1.18 |
| FNA.N.031 | North American fish | Brassy minnow | *Hybognathus hankinsoni* | Native | Wisconsin streams | 221 | 0.011 | 0.002 | 9.78 | 112.05 | 4.02 |
| FNA.N.032 | North American fish | Brook stickleback | *Culaea inconstans* | Native | Wisconsin streams | 940 | 0.017 | 0.002 | 5.93 | 52.55 | 2.38 |
| FNA.N.033 | North American fish | Brook trout | *Salvelinus fontinalis* | Native | Wisconsin streams | 460 | 0.038 | 0.003 | 3.35 | 18.04 | 1.49 |
| FNA.N.034 | North American fish | Burbot | *Lota lota* | Native | Wisconsin streams | 163 | 0.004 | 0.000 | 3.27 | 15.82 | 1.48 |
| FNA.N.035 | North American fish | Central mudminnow | *Umbra limi* | Native | Wisconsin streams | 1015 | 0.017 | 0.003 | 9.55 | 127.14 | 3.02 |
| FNA.N.036 | North American fish | Central stoneroller | *Campostoma anomalum* | Native | Wisconsin streams | 267 | 0.038 | 0.005 | 4.97 | 40.94 | 1.84 |
| FNA.N.037 | North American fish | Channel catfish | *Ictalurus punctatus* | Native | Wisconsin streams | 32 | 0.002 | 0.000 | 4.20 | 21.32 | 1.76 |
| FNA.N.038 | North American fish | Common shiner | *Luxilus cornutus* | Native | Wisconsin streams | 743 | 0.046 | 0.008 | 5.08 | 41.13 | 1.89 |
| FNA.N.039 | North American fish | Creek Chub | *Semotilus atromaculatus* | Native | Wisconsin streams | 1252 | 0.032 | 0.002 | 4.01 | 31.35 | 1.42 |
| FNA.N.040 | North American fish | Emerald shiner | *Notropis atherinoides* | Native | Wisconsin streams | 89 | 0.014 | 0.001 | 2.82 | 10.67 | 1.89 |
| FNA.N.041 | North American fish | Fantail darter | *Etheostoma flabellare* | Native | Wisconsin streams | 417 | 0.029 | 0.007 | 5.88 | 43.73 | 2.85 |
| FNA.N.042 | North American fish | Fathead minnow | *Pimephales promelas* | Native | Wisconsin streams | 437 | 0.014 | 0.002 | 9.35 | 119.63 | 3.36 |
| FNA.N.043 | North American fish | Finescale dace | *Phoxinus neogaeus* | Native | Wisconsin streams | 158 | 0.009 | 0.000 | 4.69 | 36.03 | 1.54 |
| FNA.N.044 | North American fish | Freshwater drum | *Aplodinotus grunniens* | Native | Wisconsin streams | 26 | 0.005 | 0.000 | 1.60 | 4.66 | 1.07 |
| FNA.N.045 | North American fish | Golden redhorse | *Moxostoma erythrurum* | Native | Wisconsin streams | 78 | 0.005 | 0.000 | 4.96 | 28.41 | 2.43 |
| FNA.N.046 | North American fish | Golden shiner | *Notemigonus crysoleucas* | Native | Wisconsin streams | 142 | 0.007 | 0.001 | 9.98 | 110.41 | 4.07 |
| FNA.N.047 | North American fish | Grass pickerel | *Esox americanus vermiculatus* | Native | Wisconsin streams | 40 | 0.002 | 0.000 | 2.07 | 7.33 | 0.98 |
| FNA.N.048 | North American fish | Greater redhorse | *Moxostoma valenciennesi* | Native | Wisconsin streams | 24 | 0.003 | 0.000 | 1.08 | 3.23 | 0.99 |
| FNA.N.049 | North American fish | Green Sunfish | *Lepomis cyanellus* | Native | Wisconsin streams | 404 | 0.007 | 0.000 | 5.55 | 45.15 | 2.07 |
| FNA.N.050 | North American fish | Hornyhead chub | *Nocomis biguttatus* | Native | Wisconsin streams | 453 | 0.029 | 0.002 | 3.65 | 22.12 | 1.60 |
| FNA.N.051 | North American fish | Ichthyomyzon spp. ammocoete | *Icthyomyzon spp.* | Native | North American Great Lakes tributaries | 26 | 0.007 | 0.000 | 2.59 | 9.73 | 1.55 |
| FNA.N.052 | North American fish | Iowa darter | *Etheostoma exile* | Native | Wisconsin streams | 88 | 0.003 | 0.000 | 3.17 | 15.10 | 1.41 |
| FNA.N.053 | North American fish | Johnny darter | *Etheostoma nigrum* | Native | Wisconsin streams | 951 | 0.015 | 0.001 | 4.06 | 24.57 | 1.74 |
| FNA.N.054 | North American fish | Largemouth bass | *Micropterus salmoides* | Native | Wisconsin streams | 294 | 0.004 | 0.000 | 6.23 | 59.11 | 1.95 |
| FNA.N.055 | North American fish | Largescale stoneroller | *Campostoma oligolepis* | Native | Wisconsin streams | 98 | 0.038 | 0.005 | 3.66 | 20.04 | 1.81 |
| FNA.N.056 | North American fish | Logperch | *Percina caprodes* | Native | Wisconsin streams | 183 | 0.007 | 0.000 | 6.29 | 56.31 | 2.21 |
| FNA.N.057 | North American fish | Longnose dace | *Rhinichthys cataractae* | Native | Wisconsin streams | 394 | 0.013 | 0.001 | 5.79 | 42.79 | 2.35 |
| FNA.N.058 | North American fish | Mimic shiner | *Notropis volucellus* | Native | Wisconsin streams | 39 | 0.006 | 0.000 | 3.14 | 13.16 | 1.83 |
| FNA.N.059 | North American fish | Mottled sculpin | *Cottus bairdii* | Native | Wisconsin streams | 628 | 0.027 | 0.003 | 3.68 | 20.29 | 1.86 |
| FNA.N.060 | North American fish | Northern hog sucker | *Hypentelium nigricans* | Native | Wisconsin streams | 183 | 0.010 | 0.000 | 4.78 | 33.86 | 1.74 |
| FNA.N.061 | North American fish | Northern redbelly dace | *Phoxinus eos* | Native | Wisconsin streams | 273 | 0.020 | 0.002 | 5.89 | 50.55 | 2.49 |
| FNA.N.062 | North American fish | Pearl dace | *Margariscus margarita* | Native | Wisconsin streams | 348 | 0.016 | 0.001 | 5.27 | 36.19 | 2.32 |
| FNA.N.063 | North American fish | Pumpkinseed sunfish | *Lepomis gibbosus* | Native | Wisconsin streams | 267 | 0.005 | 0.000 | 6.27 | 53.24 | 2.53 |
| FNA.N.064 | North American fish | Rainbow darter | *Etheostoma caeruleum* | Native | Wisconsin streams | 76 | 0.017 | 0.000 | 1.80 | 6.08 | 1.25 |
| FNA.N.065 | North American fish | Redbelly dace | *Phoxinus spp.* | Native | Wisconsin streams | 39 | 0.035 | 0.003 | 1.94 | 5.26 | 1.59 |
| FNA.N.066 | North American fish | Redside dace | *Clinostomus elongatus* | Native | Wisconsin streams | 75 | 0.013 | 0.000 | 2.65 | 9.92 | 1.59 |
| FNA.N.067 | North American fish | Rock Bass | *Ambloplites rupestris* | Native | Wisconsin streams | 277 | 0.007 | 0.000 | 5.37 | 46.55 | 1.71 |
| FNA.N.068 | North American fish | Rosyface shiner | *Notropis rubellus* | Native | Wisconsin streams | 92 | 0.009 | 0.000 | 2.57 | 11.33 | 1.32 |
| FNA.N.069 | North American fish | Sand shiner | *Notropis stramineus* | Native | Wisconsin streams | 114 | 0.025 | 0.007 | 9.09 | 91.31 | 3.34 |
| FNA.N.070 | North American fish | Shorthead redhorse | *Moxostoma macrolepidotum* | Native | Wisconsin streams | 159 | 0.006 | 0.000 | 3.12 | 13.46 | 1.67 |
| FNA.N.071 | North American fish | Silver redhorse | *Moxostoma anisurum* | Native | Wisconsin streams | 48 | 0.004 | 0.000 | 2.56 | 9.51 | 1.53 |
| FNA.N.072 | North American fish | Slimy sculpin | *Cottus cognatus* | Native | Wisconsin streams | 78 | 0.036 | 0.003 | 3.93 | 21.36 | 1.59 |
| FNA.N.073 | North American fish | Smallmouth bass | *Micropterus dolomieu* | Native | Wisconsin streams | 238 | 0.009 | 0.000 | 4.20 | 26.10 | 1.86 |
| FNA.N.074 | North American fish | Southern redbelly dace | *Phoxinus erythrogaster* | Native | Wisconsin streams | 184 | 0.049 | 0.011 | 3.69 | 19.07 | 2.08 |
| FNA.N.075 | North American fish | Spotfin shiner | *Cyprinella spiloptera* | Native | Wisconsin streams | 114 | 0.021 | 0.001 | 3.99 | 22.34 | 1.81 |
| FNA.N.076 | North American fish | Stonecat | *Noturus flavus* | Native | Wisconsin streams | 111 | 0.003 | 0.000 | 2.63 | 9.50 | 1.52 |
| FNA.N.077 | North American fish | Suckermouth minnow | *Phenacobius mirabilis* | Native | Wisconsin streams | 26 | 0.007 | 0.000 | 1.95 | 5.99 | 1.35 |
| FNA.N.078 | North American fish | Tadpole madtom | *Noturus gyrinus* | Native | Wisconsin streams | 51 | 0.003 | 0.000 | 3.36 | 14.09 | 1.67 |
| FNA.N.079 | North American fish | Troutperch | *Percopsis omiscomaycus* | Native | Wisconsin streams | 24 | 0.009 | 0.000 | 2.04 | 7.47 | 1.38 |
| FNA.N.080 | North American fish | Western blacknose dace | *Rhinichthys obtusus* | Native | Wisconsin streams | 803 | 0.024 | 0.002 | 3.95 | 23.78 | 1.67 |
| FNA.N.081 | North American fish | White sucker | *Catostomus commersonii* | Native | Wisconsin streams | 1184 | 0.031 | 0.002 | 3.91 | 27.50 | 1.58 |
| FNA.N.082 | North American fish | Yellow bullhead | *Ameiurus natalis* | Native | Wisconsin streams | 144 | 0.004 | 0.000 | 8.95 | 92.26 | 3.30 |
| FNA.N.083 | North American fish | Yellow perch | *Perca flavescens* | Native | Wisconsin streams | 225 | 0.010 | 0.001 | 5.65 | 40.98 | 2.64 |
| FSw.I.084 | Swedish fish | Brook trout | *Salvelinus fontinalis* | Invasive | Vasterbotten county, Sweden | 57 | 0.011 | 0.001 | 6.33 | 45.01 | 2.80 |
| FSw.N.085 | Swedish fish | Brown trout | *Salmo trutta* | Native | Vasterbotten county, Sweden | 416 | 0.018 | 0.001 | 3.11 | 15.92 | 1.57 |
| FSw.N.086 | Swedish fish | Bullhead | *Cottus gobio* | Native | Vasterbotten county, Sweden | 658 | 0.024 | 0.003 | 10.21 | 150.16 | 2.35 |
| FSw.N.087 | Swedish fish | Burbot | *Lota lota* | Native | Vasterbotten county, Sweden | 511 | 0.002 | 0.000 | 6.07 | 51.43 | 1.78 |
| FSw.N.088 | Swedish fish | Eurasian minnow | *Phoxinus phoxinus* | Native | Vasterbotten county, Sweden | 550 | 0.018 | 0.002 | 7.71 | 85.06 | 2.40 |
| FSw.N.089 | Swedish fish | European brook lamprey | *Lampetra planeri* | Native | Vasterbotten county, Sweden | 177 | 0.003 | 0.000 | 10.45 | 125.44 | 3.00 |
| FSw.N.090 | Swedish fish | European perch | *Perca fluviatilis* | Native | Vasterbotten county, Sweden | 138 | 0.003 | 0.000 | 4.26 | 23.46 | 2.04 |
| FSw.N.091 | Swedish fish | Grayling | *Thymallus thymallus* | Native | Vasterbotten county, Sweden | 352 | 0.002 | 0.000 | 7.99 | 81.38 | 2.30 |
| FSw.N.092 | Swedish fish | Northern pike | *Esox lucius* | Native | Vasterbotten county, Sweden | 343 | 0.001 | 0.000 | 6.24 | 50.71 | 1.88 |
| M.I.093 | Mussel | Zebra mussel | *Dresseina polymorpha* | Invasive | Europe | 56 | 0.019 | 0.001 | 3.30 | 14.91 | 1.34 |
| M.I.094 | Mussel | Zebra mussel | *Dresseina polymorpha* | Invasive | North America | 19 | 0.247 | 0.090 | 1.35 | 3.51 | 1.22 |
| Pl.I.095 | Plant | Eurasian water milfoil | *Myriophyllum spicatum* | Invasive | Wisconsin Lakes | 100 | 0.219 | 0.049 | 1.23 | 3.97 | 1.01 |
| Pl.I.096 | Plant | Curly leaf pondweed | *Potamogeton crispus* | Invasive | Wisconsin Lakes | 63 | 0.098 | 0.022 | 2.57 | 9.96 | 1.52 |
| Pl.N.097 | Plant | Coontail | *Ceratophyllum demersum* | Native | Wisconsin Lakes | 161 | 0.258 | 0.072 | 1.22 | 3.40 | 1.04 |
| Pl.N.098 | Plant | Muskgrass | *Chara spp.* | Native | Wisconsin Lakes | 175 | 0.306 | 0.070 | 0.72 | 2.47 | 0.86 |
| Pl.N.099 | Plant | Canadian waterweed | *Elodea canadensis* | Native | Wisconsin Lakes | 140 | 0.171 | 0.033 | 1.32 | 3.98 | 1.06 |
| Pl.N.100 | Plant | Quillwort | *Isoetes spp.* | Native | Wisconsin Lakes | 50 | 0.045 | 0.005 | 3.20 | 14.73 | 1.51 |
| Pl.N.101 | Plant | Shortspike water milfoil | *Myriophyllum sibiricum* | Native | Wisconsin Lakes | 117 | 0.090 | 0.010 | 1.83 | 6.59 | 1.09 |
| Pl.N.102 | Plant | Slender water milfoil | *Myriophyllum tenellum* | Native | Wisconsin Lakes | 50 | 0.058 | 0.005 | 2.89 | 13.93 | 1.22 |
| Pl.N.103 | Plant | Nodding waternymph | *Najas flexilis* | Native | Wisconsin Lakes | 165 | 0.175 | 0.044 | 1.56 | 4.58 | 1.20 |
| Pl.N.104 | Plant | Stonewort | *Nitella spp.* | Native | Wisconsin Lakes | 132 | 0.125 | 0.018 | 1.66 | 6.19 | 1.08 |
| Pl.N.105 | Plant | Varigated yellow pond lily | *Nuphar variegata* | Native | Wisconsin Lakes | 135 | 0.047 | 0.003 | 1.85 | 6.33 | 1.11 |
| Pl.N.106 | Plant | Fragrant waterlily | *Nymphaea odorata* | Native | Wisconsin Lakes | 164 | 0.071 | 0.007 | 3.20 | 19.24 | 1.23 |
| Pl.N.107 | Plant | Largeleaf pondweed | *Potamogeton ampifolius* | Native | Wisconsin Lakes | 120 | 0.089 | 0.008 | 2.53 | 13.63 | 1.02 |
| Pl.N.108 | Plant | Small pondweed | *Potamogeton pusillus* | Native | Wisconsin Lakes | 129 | 0.097 | 0.012 | 1.66 | 5.55 | 1.15 |
| Pl.N.109 | Plant | Flatstem pondweed | *Potamogeton zosteriformis* | Native | Wisconsin Lakes | 119 | 0.114 | 0.016 | 2.06 | 9.10 | 1.11 |
| Pl.N.110 | Plant | Wild celery | *Vallisneria americana* | Native | Wisconsin Lakes | 128 | 0.120 | 0.014 | 1.20 | 3.92 | 0.97 |
| Pr.I.111 | Prawn | Tahitian prawn | *Macrobrachium lar* | Invasive | Hawaiian Streams | 71 | 0.255 | 0.054 | 1.73 | 5.26 | 0.92 |
| Pr.N.112 | Prawn | ‘Ōpae ‘oeha‘a | *Macrobrachium grandimamus* | Native | Hawaiian Streams | 21 | 0.106 | 0.004 | 1.09 | 3.40 | 0.61 |
| S.I.113 | Snail | Chinese mystery snail | *Bellamya chinensis* | Invasive | Wisconsin Lakes | 22 | 0.011 | 0.001 | 2.61 | 8.91 | 2.15 |
| S.N.114 | Snail | Mud amnicola | *Amnicola limosa* | Native | Wisconsin Lakes | 36 | 0.140 | 0.056 | 2.44 | 8.09 | 1.68 |
| S.N.115 | Snail | Ponted campeloma | *Campeloma decisum* | Native | Wisconsin Lakes | 20 | 0.003 | 0.000 | 1.54 | 4.10 | 1.29 |
| S.N.116 | Snail | Flexed gyro snail | *Gyraulus deflectus* | Native | Wisconsin Lakes | 26 | 0.009 | 0.000 | 1.72 | 6.46 | 0.96 |
| S.N.117 | Snail | Ash gyro snail | *Gyraulus parvus* | Native | Wisconsin Lakes | 28 | 0.006 | 0.000 | 2.66 | 10.60 | 1.40 |
| S.N.118 | Snail | Two-ridge rams horn | *Helisoma anceps* | Native | Wisconsin Lakes | 29 | 0.012 | 0.000 | 1.62 | 4.51 | 1.37 |
| S.N.119 | Snail | NA | *Helisoma campanulata* | Native | Wisconsin Lakes | 30 | 0.006 | 0.000 | 3.70 | 17.67 | 1.68 |
| S.N.120 | Snail | Boreal marstonia | *Marstonia lustrica* | Native | Wisconsin Lakes | 28 | 0.056 | 0.014 | 2.45 | 7.28 | 2.12 |
| S.N.121 | Snail | Tadpole physa | *Physa gyrina* | Native | Wisconsin Lakes | 29 | 0.007 | 0.000 | 1.81 | 5.20 | 1.53 |

Literature Cited

1. Naddafi R, Blenckner T, Eklov P, Pettersson K (2011) Physical and chemical properties determine zebra mussel invasion success in lakes. Hydrobiologia 669: 227-236.

2. Bailey RC, Grapentine L, Stewart TJ, Schaner T, Chase ME, et al. (1999) Dreissenidae in Lake Ontario: Impact assessment at the whole lake and Bay of Quinte spatial scales. Journal of Great Lakes Research 25: 482-491.

3. Ricciardi A, Whoriskey FG, Rasmussen JB (1997) The role of the zebra mussel (*Dreissena* *polymorpha*) in structuring macroinvertebate communities on hard substrata. Can J Fish Aquat Sci 54: 2596-2608.

4. Mercer JL, Fox MG, Metcalfe CD (1999) Changes in benthos and three littoral zone fishes in a shallow, eutrophic Ontario lake following the invasion of the zebra mussel (*Dreissena polymorpha*). Lake and Reservoir Management 15: 310-323.

5. Karatayev AY, Burlakova LE (2008) Potential effects of zebra mussels in the Madison lakes. Wisconsin DNR Aquatic Invasive Species Grants Program. 67 p.

6. Nystrom P, Stenroth P, Holmqvist N, Berglund O, Larsson P, et al. (2006) Crayfish in lakes and streams: Individual and population responses to predation, productivity, and substratum availability. Freshwater Biol 51: 2096-2113.

7. Larson ER, Olden JD (2012) Crayfish occupancy and abundance in lakes of the Pacific northwest, USA. Freshwater Science: 94-107.

8. Kornis MS, Sharma S, Vander Zanden MJ (2013) Invasion success and impact of an invasive fish, round goby, in Great Lakes tributaries. Diversity and Distributions 19: 184-198.

9. Slade JW, Adams JV, Christie GC, Cuddy DW, Fodale MF, et al. (2003) Techniques and methods for estimating abundance of larval and metamorphosed sea lampreys in Great Lakes tributaries, 1995-2001. Journal of Great Lakes Research 29 (Supplement 1): 137-151.

10. Hansen GJA, Jones ML (2008) A rapid assessment approach to prioritizing streams for control of Great Lakes sea lampreys (*Petromyzon* *marinus*): A case study in adaptive management. Can J Fish Aquat Sci 65: 2471-2484.

11. Higashi GR, Nishimoto RT. The point quadrat method: A rapid assessment of Hawaiian streams. In: Evenhuis NL, Fitzsimons JM, editors; 2007; Bishop Museum Bulletin in Cultural and Environmental Studies. pp. 305-313.

12. Solomon CT, Olden JD, Johnson PTJ, Dillon RT, Vander Zanden MJ (2010) Distribution and community-level effects of the chinese mystery snail (*Bellamya chinensis*) in northern Wisconsin lakes. Biol Invasions 12: 1591-1605.

13. Mikulyuk AM, Hauxwell J, Rasmussen P, Knight S, Wagner KI, et al. (2010) Testing a methodology for assessing plant communities in temperate inland lakes. Lake and Reservoir Management 26: 54-62.

14. Capelli GM (1982) Displacement of northern Wisconsin crayfish by *Orconectes rusticus* (Girard). Limnol Oceanogr 27: 741-745.

15. Capelli GM, Magnuson JJ (1983) Morphoedaphic and biogeographic analysis of crayfish distribution in northern Wisconsin. J Crustacean Biol 3: 548-564.

16. Wilson KA, Magnuson JJ, Lodge DM, Hill AM, Kratz TK, et al. (2004) A long-term rusty crayfish (*Orconectes* *rusticus*) invasion: Dispersal patterns and community change in a north temperate lake. Can J Fish Aquat Sci 61: 2255–2266.

17. Lodge DM, Kershner MW, Aloi JE, Covich AP (1994) Effects of an omnivorous crayfish (*Orconectes rusticus*) on a freshwater littoral food web. Ecology 75: 1265–1281.

18. McCarthy JM, Hein CL, Olden JD, Jake Vander Zanden M (2006) Coupling long-term studies with meta-analysis to investigate impacts of non-native crayfish on zoobenthic communities. Freshwater Biol 51: 224–235.

19. Abrahamsson S (1973) The crayfish *Astacus astacus* in sweden and the introduction of the american crayfish *Pacifastacus leniusculus*. In: Abrahamsson S, editor. Freshwater crayfish papers from the first international symposium on freshwater crayfish, Austria, 1972. Lund, Sweden: Studentlitteratur. pp. 27-40.

20. Nystrom P, Strand J (1996) Grazing by a native and an exotic crayfish on aquatic macrophytes. Freshwater Biol 36: 673-682.

21. Nyström P, Brönmark C, Graneli W (1999) Influence of an exotic and a native crayfish species on a littoral benthic community. Oikos 85: 545-553.

22. Crawford L, Yeomans WE, Adams CE (2006) The impact of introduced signal crayfish *Pacifastacus leniusculus* on stream invertebrate communities. Aquatic Conservation: Marine and Freshwater Ecosystems 16: 611-621.

23. Yamamoto MN, Tagawa AW (2000) Hawai'i's native and exotic freshwater animals. Honolulu, HI, USA: Mutual Publishing.

24. Maciolek JA (1984) Exotic fishes in Hawaii and other islands of oceania. In: Courtenay Jr. WR, Stauffer Jr. JR, editors. Distribution, biology, and management of exotic fishes. Baltimore, MD: The Johns Hopkins University Press. pp. 131-161.

25. Mundy BC (2005) Checklist of the fishes of the Hawaiian archipelago. Bishop Museum Bulletins in Zoology 6: 704 pp.

26. Brock VE (1960) The introduction of aquatic animals into Hawaiian waters. International Revue de Gesamten Hydrobiologie 45: 463-480.

27. Devick WS (1991) Patterns of introductions of aquatic organisms into Hawaiian freshwater habitats. New directionsin research, management and conservation of Hawaiian freshwater stream ecosystems. Hawaii Department of Land and Natural Resources: Proceedings of the 1990 symposium on frehwater stream biology and fisheries management. pp. 189-213.

28. MacCrimmon HR, Marshall TL, Gots BL (1970) World distribution of brown trout, *Salmo trutta*: Further observations. J Fish Res Board Can 27: 811-818.

29. Crowl TA, Townsend CR, McIntosh AR (1992) The impact of introduced brown and rainbow trout on native fish: The case of Australasia. Reviews in Fish Biology and Fisheries 2: 217-241.

30. Townsend CR (1996) Invasion biology and ecological impacts of brown trout *Salmo trutta* in New zealand. Biol Conserv 78: 13-22.

31. McHugh P, Budy P (2005) An experimental evaluation of competitive and thermal effects on brown trout (*Salmo trutta*) and Bonneville cutthroat trout (*Oncorhynchus clarkii utah*) performance along an altitudinal gradient. Can J Fish Aquat Sci 62: 2784-2795.

32. DeVaney SC, McNyset KM, Williams JB, Peterson AT, Wiley EO (2009) A tale of four "carp'': Invasion potential and ecological niche modeling. Plos One 4: E5451; Doi 5410.1371/Journal.Pone.0005451.

33. Zambrano L, Scheffer M, Martinez-Ramos M (2001) Catastrophic response of lakes to benthivorous fish introduction. Oikos 94: 344-350.

34. Miller SA, Crowl TA (2005) Effects of common carp (*Cyprinus carpio*) on macrophytes an invertebrate communities in a shallow lake. Freshwater Biol 51: 85-94.

35. Matsuzaki S-iS, Usio N, Takamura N, Washitani I (2008) Contrasting impacts of invasive engineers on freshwater ecosystems: An experiment and meta-analysis. Oecologia 158: 673-686.

36. MacCrimmon HR (1971) World distribution of rainbow trout (*Salmo gairdneri*). J Fish Res Board Can 28: 663-704.

37. Allendorf FW, Leary RF (1988) Conservation and distribution of genetic variation in a polytypic species, the cutthroat trout. Conserv Biol 2: 170-184.

38. Nyström P, Svensson O, Lardner B, Brönmark C, Granéli W (2001) The influence of multiple introduced predators on a littoral pond community. Ecology 82: 1023-1031.

39. Charlebois PM, Marsden JE, Goettel RG, Wolfe RK, Jude DJ, et al. (1997) The round goby, *Neogobius melanostomus* (Pallas): A review of European and North American literature. Champaign, IL, USA: Illinois Natural History Survey.

40. Poos M, Dextrase AJ, Schwalb AN, Ackerman JD (2010) Secondary invasion of the round goby into high diversity Great Lakes tributaries and species at risk hotspots: Potential new concerns for endangered freshwater species. Biol Invasions 12: 1269-1284.

41. Kornis MS, Mercado-Silva N, Vander Zanden MJ (2012) Twnty years of invasion: A review of round goby *Neogobius melanostomus* biology, spread, and ecological implications. Journal of Fish Biology 80: 235-285.

42. Corkum LD, Sapota MR, Skora KS (2004) The round goby, *Neogobius melanostomus,* a fish invader on both sides of the Atlantic ocean. Biol Invasions 6: 173-181.

43. Applegate VC (1950) Natural history of the sea lamprey *(Petromyzon marinus*) in Michigan. US Fish and Wildlife Service. 237 p.

44. Fetterolf Jr CM (1980) Why a Great Lakes fishery commission and why a sea lamprey international symposium? Can J Fish Aquat Sci 37: 1588-1593.

45. Pearce WA, Braem RA, Dustin SM, Tibbles JJ (1980) Sea lamprey (*Petromyzon* *marinus*) in the lower Great Lakes. Can J Fish Aquat Sci 37: 1802-1810.

46. Smith BR, Tibbles JJ (1980) Sea lamprey (*Petromyzon* *marinus*) in lakes Huron, Michigan, and Superior: History of invasion and control, 1936-78. Can J Fish Aquat Sci 37: 1780-1801.

47. MacCrimmon HR, Campbell JS (1969) World distribution of brook trout, *Salvelinus fontinalis*. J Fish Res Board Can 26: 1699-1725.

48. Spens J, Alanara A, Eriksson L-O (2007) Nonnative brook trout (*Salvelinus fontinalis*) and the demise of native brown trout (*Salmo trutta*) in northern boreal lakes: Stealthy, long-term patterns? Can J Fish Aquat Sci 64: 654-664.

49. Ohlund G, Nordwall F, Degerman E, Eriksson T (2008) Life history and large-scale habitat use of brown trout (*Salmo trutta*) and brook trout (*Salvelinus fontinalis*) - implications for species replacement patterns. Can J Fish Aquat Sci 65: 633-644.

50. Stańczykowska A (1977) Ecology of *Dreissena* *polymorpha* (Pall.)(bivalvia) in lakes. Polish Archives of Hydrobiology 24: 461-530.

51. Griffiths RW, Schloesser DW, Leach JH, Kovalak WP (1991) Distribution and dispersal of the zebra mussel (*Dreissena* *polymorpha* ) in the Great Lakes region. Can J Fish Aquat Sci 48: 1381-1388.

52. Ludyanskiy ML, McDonald D, MacNeill D (1993) Impact of the zebra mussel, a bivalve invader. Bioscience 43: 533-544.

53. Strayer DL (2008) Twenty years of zebra mussels: Lessons from the mollusk that made headlines. Front Ecol Environ 7: 135-141.

54. Higgins SN, Vander Zanden MJ (2010) What a difference a species makes: A meta-analysis of Dreissenid mussel impacts on freshwater ecosystems. Ecol Monogr 80: 179-196.

55. (2012) Wisflora: Vascular plant species online database. University of Wisconsin-Madison, Madison, Wisconsin: Wisconsin State Herbarium.

56. Bolduan BR, Van Eeckhout GC, Quade HW, Gannon JE (1994) *Potamogeton crispus* - the other invader. Lake and Reservoir Management 10: 113-125.

57. Nichols SA, H. SB (1986) Ecological life histories of the three aquatic nuisance plants, *Myriophyllum spicatum*, *Potamogeton crispus* and *Elodea canadensis* Hydrobiolgia 131: 3-21.

58. Boylen CW, Eichler LW, Madsen JD (1999) Loss of native aquatic plant species in a community dominated by Eurasian watermilfoil. Hydrobiolgia 415: 207-211.

59. Madsen JD (2001) Epiphytic macroinvertebrates along a gradient of Eurasian watermilfoil cover. Journal of Aquatic Plant Management 39: 67-72.

60. Cheruvelil KS, Soranno PA, Madsen JD, Roberson MJ (2002) Plant architecture and epiphytic macroinvertebrate communities: The role of an exotic dissected macrophyte. J N Am Benthol Soc 21: 261-277.

61. Johnson PTJ, Olden JD, Solomon CT, Vander Zanden MJ (2009) Interactions among invaders: Community and ecosystem effects of multiple invasive species in an experimental aquatic system. Oecologia 159: 161-170.
